# Doctors Should Not Be so Modest

**Published:** 1896-09

**Authors:** 


### DOCTORS SHOULD NOT BE SO MODEST.

I know that there is a general feeling among physicians of the better sort that conspicuous interest in public affairs may be misconstrued and looked upon as in some sort a means of professional advertisement. And one can not choose but to appreciate and admire the sensitiveness and high sense of honor of which this sentiment is born. But, after all, there are greater misfortunes in life than being misunderstood, and I think that the fine feeling which leads the physician so often to waive the privileges of social and public life in the interest of what he conceives to be professional ethics is capable of a richer fruitage yet, in the defiance of misconstruction, when impelled to whatever performance of public duty he can justify to himself.—*Medical Record.*

